# Assessment of the scale, coverage and outcomes of the Avahan HIV prevention program for female sex workers in Tamil Nadu, India: is there evidence of an effect?

**DOI:** 10.1186/1471-2458-11-S6-S3

**Published:** 2011-12-29

**Authors:** S Thilakavathi, K Boopathi, CP Girish Kumar, A Santhakumar, R Senthilkumar, C Eswaramurthy, V Ilaya Bharathy, L Ramakrishnan, G Thongamba, R Adhikary, R Paranjape

**Affiliations:** 1 National Institute of Epidemiology (ICMR), Second Main Road, TNHB, Ayapakkam, Chennai 600 077, India; 2 FHI India, H-5 (Ground Floor), Green Park Extension, New Delhi 110016, India; 3 National AIDS Research Institute (ICMR), T 71-1A/2, M.I.D.C., Telco Road, Bhosari, Pune - 411 026, India

## Abstract

**Background:**

Avahan, the India AIDS Initiative, a large-scale HIV prevention program, using peer-mediated approaches and STI services, was implemented for high-risk groups for HIV in six states in India. This paper describes the assessment of the program among female sex workers (FSWs) in the southern state of Tamil Nadu.

**Methods:**

An analytical framework based on the Avahan impact evaluation design was used. Routine program monitoring data, two rounds of cross-sectional biological and behavioural surveys among FSWs in 2006 (Round 1) and 2009 (Round 2) and quality assessments of clinical services for sexually transmitted infections (STIs) were used to assess trends in coverage, condom use and prevalence of STIs, HIV and their association with program exposure. Logistic regression analysis was used to examine trends in intermediate outcomes and their associations with intervention exposure.

**Results:**

The Avahan program in Tamil Nadu was scaled up and achieved monthly reported coverage of 79% within four years of implementation. The cross-sectional survey data showed an increasing proportion of FSWs being reached by Avahan, 54% in Round 1 and 86% in Round 2 [AOR=4.7;p=0.001]. Quality assessments of STI clinical services showed consistent improvement in quality scores (3.0 in 2005 to 4.5 in 2008). Condom distribution by the program rose to cover all estimated commercial sex acts. Reported consistent condom use increased between Round 1 and Round 2 with occasional (72% to 93%; AOR=5.5; p=0.001) and regular clients (68% to 89%; AOR=4.3; p=0.001) while reactive syphilis serology declined significantly (9.7% to 2.2% AOR=0.2; p=0.001). HIV prevalence remained stable at 6.1% between rounds. There was a strong association between Avahan exposure and consistent condom use with commercial clients; however no association was seen with declines in STIs.

**Conclusions:**

The Avahan program in Tamil Nadu achieved high coverage of FSWs, resulting in outcomes of improved condom use, declining syphilis and stabilizing HIV prevalence. These expected outcomes following the program logic model and declining HIV prevalence among general population groups suggest potential impact of high risk group interventions on HIV epidemic in Tamil Nadu.

## Background

India, with many concentrated HIV epidemics [1], had an estimated 2.5 million (1.75–3.15 million) people living with HIV in 2006 [2-4]. The key drivers of the epidemic are the size of the sex worker population and frequency of commercial sex, mainly heterosexual, similar to many other Asian countries [5,6]. Targeted interventions among female sex workers (FSWs) and their clients have been effective in containing the HIV in concentrated epidemics elsewhere [7-11] and have been a key strategy in India’s response [12].

Avahan, the India AIDS Initiative, a large-scale HIV prevention intervention targeting high-risk groups, including FSWs, began in late 2003 aiming to scale up high-risk group interventions to contain the HIV epidemic at the population level [13]. Avahan delivered a package of proven prevention services, addressing proximal and distal determinants of HIV risk quickly, across a large area with high coverage [14].

**Figure 1:**
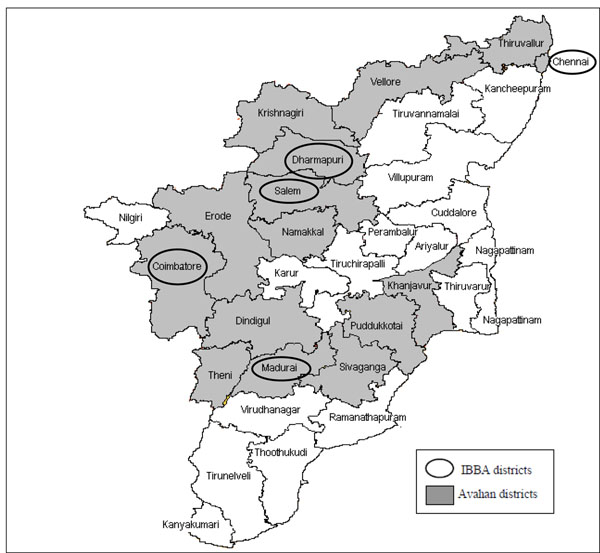
Tamil Nadu District Map with Avahan Intervention and IBBA districts

The Avahan program in Tamil Nadu covered FSWs, men who have sex with men and transgender populations, men at sex worker solicitation points (“hot-spots”) and long-distance truckers and was launched in 14 out of 32 districts in Tamil Nadu in consultation with the state government in October 2004. Figure 1 shows the location of Avahan districts in Tamil Nadu. Avahan interventions with FSWs established or strengthened existing programs for FSWs including peer-based outreach education, promotion and distribution of condoms, establishing program-linked clinics to manage sexually transmitted infections (STIs) and community mobilization in selected districts. In most Avahan districts, Avahan was the only provider of FSW interventions. Table 1 shows size estimates of FSWs in Avahan districts, Avahan’s intended coverage and details of prior interventions in these districts. From 2007-2008, as part of third phase of the Indian National AIDS Control Program, geographic areas were allocated by the major state partners for implementation of targeted interventions, so that there was only one provider per district to avoid duplication of efforts [15].

**Table 1:** District-level Avahan intended coverage and history of intervention in Tamil Nadu

Districts	District population^1^ (thousands)	Estimated size of FSW in districts^2^	Avahan intended coverage^3^	History / type of intervention coverage*	IBBA sample	
					
					R-I	R-II
Chennai	2,219	17,392	17%	Minority- Transferred to NACO / Chennai AIDS Prevention Control Society in April 2008	410	397
Coimbatore	2,176	2000	100%	Not first but solo	410	400
Dharmapuri	1,473	3000	100%	First and solo	408	406
Dindigul	968	1500	100%	Not first but solo		
Erode	1,309	1500	100%	First and solo		
Krishnagiri^*^	-	3500	100%	First and solo		
Madurai	1,303	7000	100%	Not first but solo	402	396
Namakkal	759	1600	100%	Not first but solo		
Salem	1,563	5100	100%	First and solo	402	407
Thanjavur	1,096	1500	100%	Not first but solo		
Theni	553	1750	100%	Not first but solo		
Thiruvallur	1,397	4000	100%	First and solo		
Vellore	1,741	2500	100%	Not first but solo		

**Source: 1-**Census 2001; **2-**District mapping data **3-**Avahan CMIS 2009

**First and solo: 1)** Prior to Avahan (i.e. 2003), there was no intervention for FSW; **2)** Currently Avahan is the only intervention in the district.

**Not first but major:** Prior to Avahan (i.e. 2003), FSW intervention existed in the districts and currently Avahan covers more than 50% of the mapped FSW population in the district

**Not first but equal:** Prior to Avahan (i.e. 2003), FSW intervention existed in the districts and currently Avahan covers 30 - 50% of the mapped FSW population in the district

**Not first and only clinical services:** Prior to Avahan (i.e. 2003), FSW intervention existed in the districts and Avahan provides only clinical services in the district

*Separated from Dharmapuri in 2004-05

The Avahan assessment followed the program logical model step-by-step to: a) assess scale-up and coverage of FSWs in the selected sites; b) measure declines in HIV risk behaviours; c) measure trends in of STI and HIV prevalence among FSWs; d) document trends in HIV prevalence among the general population; and e) assess possible association of changes to Avahan interventions. Therefore, if scale and coverage were achieved, the assessment design proposed to measure the effectiveness of interventions in achieving condom use and averting HIV infections among FSWs and the general population [16]. The conceptual framework developed by Habicht and Victoria for large-scale public health program evaluations using the concepts of adequacy and plausibility was adopted for the Avahan assessment [17].

This paper describes evidence of the impact of the Avahan program for FSWs in Tamil Nadu by systematically addressing data by each level of the program logic model including coverage, intermediate outcomes and their association with exposure to the Avahan program within the context of state-level HIV prevention efforts.

## Methods

### Analytical framework for assessment

The analytical framework for this assessment was developed drawing directly from the Avahan program evaluation design [16]. This framework addresses the assessment questions step-by-step following the logical sequence of program implementation (process and output indicators), intermediate outcomes and contributions of Avahan. The complete framework and its details are presented in another paper in this supplement [18]. The specific aims in this analysis were to assess: (a) the scale, intensity (based on availability and utilization of services), and quality of Avahan coverage; (b) the intermediate outcome of consistent condom use; (c) changes in prevalence of STIs and new HIV infections; and (d) the association of Avahan exposure to changes in condom use and STI prevalence.

### Data sources

The present analysis used Avahan routine program monitoring data, two rounds of cross-sectional surveys conducted among FSWs (termed as Integrated Behavioural and Biological Assessments – IBBAs), condom sales data from social marketing efforts, and external quality assessments conducted in Avahan STI clinics.

#### a. Avahan routine program monitoring data

Avahan developed a computerized management information system (CMIS) through the course of program implementation [19,20], which provided data on program inputs, infrastructure, outreach and clinical service utilization. STI clinic data were tracked at the individual level through NGO issued identification numbers. Data from these sources were used to examine trends in coverage and uptake of services from early in the program through March 2009 [19]. The scope of this analysis included 14 of 32 districts of Tamil Nadu where Avahan interventions had been implemented from 2005 to 2009. Some districts were transferred to NACO between 2007 and 2008 and, by January 2009, Avahan districts numbered 12, including some districts transferred from NACO to the Avahan program based on the state plan for implementing phase III of the Indian National AIDS Control Plan [15].

#### b. Integrated Behavioural and Biological Assessments

Two rounds of cross-sectional IBBAs were undertaken among FSWs [21]. Round 1 was conducted between March and December 2006 and Round 2 between March and September 2009, in five purposively sampled Avahan districts: Chennai, Coimbatore, Dharmapuri-Krishnagiri combined, Madurai and Salem, based on the large size of FSW population and to represent the different socio-cultural regions of the state [21]. The same probability based sampling methodology was used in both rounds [22]. Field work was conducted by research agencies under the guidance and supervision of the implementing State Indian Council of Medical Research (ICMR) Institute-National Institute of Epidemiology (NIE). The National AIDS Research Institute (NARI) was the national coordinating center. The international agency, FHI, provided technical assistance for conducting IBBA. Ethical clearances were obtained prior to surveys from Protection of Human Subjects Committee of FHI and the ethics committee of the NIE-ICMR. Written informed consent was obtained from respondents. Full details of the IBBA methodology have been published previously [21].

#### c. STI clinical quality monitoring assessments

A central STI capacity building team, led by FHI, was responsible for ensuring high quality standardized STI services [23] and developed a clinical quality monitoring tool [24]. These quality monitoring assessments of STI services were conducted quarterly by an external team in Tamil Nadu between 2005 and 2009. A detailed methodology of these assessments has been described elsewhere [24]. Total scores were calculated and a correlation matrix was used to examine changes in scores over time using STATA 11® (Stata Corporation, College Station, TX).

### Operational definitions and assumptions

Coverage was defined on the basis of availability and utilization of HIV prevention services [19] for FSWs. The adequacy of coverage was defined based on the Avahan target for saturated coverage, set at 80% of the estimated denominator of the target population in the intervention districts. This denominator of FSWs determined in March 2009 was used for the entire time period of analysis in this assessment [18].

The Avahan target for outreach contacts was a minimum of one contact per month, whereas the target for clinic visits was once per quarter (about 33% of the estimated denominator per month) for STI consultations [25]. The CMIS data were validated (‘evaluated coverage’) by comparing with corresponding IBBA data [18].

Intensity was defined as the frequency of exposure to the Avahan program services measured based on indicators listed in the framework [18]. Staffing to achieve intensity program was measured using two Avahan CMIS indicators [18] against the target ratio of 1:50) [25]. Free condom distribution to FSWs by the program was tracked yearly in Avahan CMIS and annual condom sales data from condom social marketing program [26]. These data and IBBA data were used to analyse for gap between commercial sex acts and condoms provided, per FSW [18]. Data on condom sources from IBBA were used to validate trends shown in the CMIS.

Self-reported condom use from the two rounds of IBBAs was used to assess changes in condom use with commercial and non-commercial partners of FSWs. Consistent condom was defined as condom use every time and no reported unprotected sex acts defined as condom use every time with both occasional and regular clients.

Changes in STI prevalence were measured based on tests conducted on blood and urine samples collected from FSWs during IBBAs. Biological tests included syphilis serology using Rapid Plasma Reagin (RPR) and confirmatory *Treponema pallidum* Hemagglutination Assay (TPHA), and nucleic acid amplification (Gen-Probe APTIMA COMBO 2) of urine samples for chlamydial and gonococcal prevalence [21]. Any positive RPR confirmed with TPHA was defined as reactive syphilis or lifetime syphilis; whereas RPR yielding titres ≥1:8 or more and positive TPHA were defined as active or high-titre syphilis.

Data on HIV prevalence were assessed from HIV serology from two rounds of IBBA. HIV positivity was determined using a two-test algorithm using enzyme immunoassay (J. Mitra and Genedia- EIAs) [21]. As a proxy for new HIV infections, HIV prevalence among newer FSWs, those who entered sex work in the last year, and among FSWs aged from 18 to 20 years (FSWs younger than 18 years were excluded from the survey), were examined.

A composite indicator of exposure, having received any of the three core program services was used for the analysis [18]. Pooled IBBA data from the two rounds were used to examine the association between exposure to Avahan interventions and condom use and STIs.

#### Data management and statistical methods

Double-data entry of district-level datasets was conducted using CSPro Software (U.S. Census Bureau, Washington DC) for both rounds of the IBBAs. SPSS 14.0® (IBM, Somers NY) statistical software was used for data analysis. District-level data of each round were merged to generate state-level datasets for Round 1 and Round 2. For some analyses, these merged data from Round 1 and 2 were aggregated to obtain pooled data. Appropriate weights at the district-level and state-level datasets for Rounds 1 and 2 were calculated and used for analysis [21]. Bi-variate and multivariate analyses were conducted using the complex samples module in SPSS. The Wald Chi-square test was used to assess significant changes in characteristics among FSWs between the two rounds of IBBAs. Multivariate logistic regression was used to assess significant changes in: (a) exposure measures; (b) condom use outcomes with different partner types; and (c) prevalence of STIs and HIV, between the two IBBA rounds. The merged dataset was used to study the association between exposure to Avahan services and having any STI (gonorrhoea, chlamydia or high-titre syphilis) and consistent condom use with commercial and non-commercial partners. Profile variables found to be significant in bivariate analysis between two surveys were adjusted (controlled) in logistic regression models to generate adjusted odds ratios (AORs). Associations were considered significant for p-values lower than 0.05.

## Results

A total of 4,038 FSWs (2,032 in Round 1 and 2,006 in Round 2) were sampled in the two rounds of IBBAs. The number of sampled FSWs in the five districts is shown in Table 1. The profile characteristics of FSWs in Rounds 1 and 2 [27] is provided in Table 2. About 15.5% of FSWs in Round 2 reported that they had participated in Round 1.

**Table 2:** Socio-demographic and sex work characteristics of female sex workers in Tamil Nadu in Rounds 1 and 2 of IBBA

Characteristics	Groups	RI (%) N=2032	RII (%) N=2006	P Value (Wald Chi-square)
**Current age (years)**	**<25**	13.6	9.9	0.033
	**25-29**	22.0	19.1	
	**30-34**	22.3	20.8	
	**35-39**	25.8	27.6	
	**40+**	16.3	22.6	

**Mean**		32.3	33.8	

**Literacy**	**Illiterate**	57.7	41.8	<0.001
	**Literate**	42.3	58.2	

**Marital status**	**Unmarried**	3.2	13.1	<0.001
	**Married**	72.5	57.5	
	**Divorced/ separated / widowed**	24.3	29.4	

**Additional income**	**None**	41.8	22.5	<0.001
	**Yes**	58.2	77.5	

**Locality status**	**Yes**	90.2	91.6	0.488
	**No**	9.8	8.5	

**Sex work in city <2 years**	**Yes**	19.8	10.9	<0.001
	**No**	80.1	89.1	

**Age at first sex**	**< 15**	12.7	10.2	0.064
	**15+**	87.1	89.8	

**Mean**		17.8	17.8	

**Age when started sex work**	**<20**	9.5	6.8	0.242
	**20-24**	25.1	24.2	
	**25-29**	29.2	28.6	
	**30+**	36.3	40.4	

**Duration in sex work**	**0-1**	17.3	11.5	0.005
	**2 to 4**	42.3	38.5	
	**4 to 9**	22.3	26.7	
	**10+**	18.0	23.2	

Usual place of solicitation/ Typology of sex work*	**Non-street based**	8.7	4.5	0.049
	**Street**	91.3	95.5	

**Usual place of entertainment**	**Home**	66.2	71.6	0.235
	**Brothel/ lodge**	20.8	17.9	
	**Street**	13.0	10.5	

**Clients per week**	**0 to 4**	36.0	34.9	0.112
	**5 to 9**	44.5	49.7	
	**10+**	19.5	15.6	

**Mean volume of clients**		6.5	5.8	

**Regular partner**	**Yes**	76.8	75.9	0.769
	**No**	23.3	24.1	

*Refers to the type of sex work practiced by FSWs; non-street based sex work refers to FSWs who solicit clients in fixed venues, such as homes, brothels or lodges; whereas the street based sex work is when FSWs solicit their clients from public places such as bus stands, shops, markets, parks etc.

### Adequacy of Avahan program coverage

#### Scale of coverage

**Figure 2:**
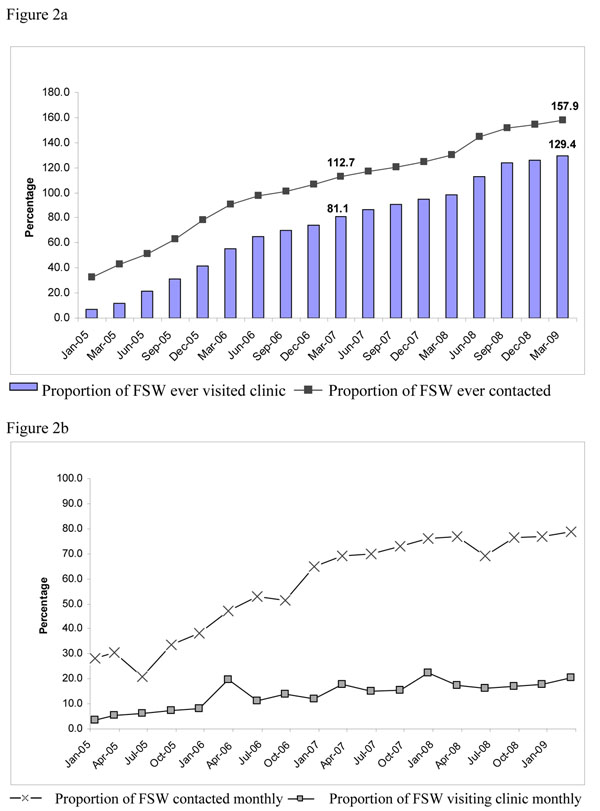
a) Proportion of FSW ever contacted and ever visited program clinic in Tamil Nadu (Avahan CMIS 2005-2009). b) Proportion of FSWs contacted and visiting clinic in a month in Maharashtra (Avahan CMIS: 2005 - 2009)

The Avahan program in Tamil Nadu was scaled up from 11 districts in July 2006 to 14 by March 2007 and stabilized at 12 districts through 24 local NGOs. The intended coverage for Avahan was 34,950 FSWs as of March 2009 (not including Chennai which was transferred to the State program in April 2008). Table 1 provides size estimates by each Avahan district, the intended coverage of Avahan and the history of prior interventions. A total of 55,000 individual FSWs (158%) were contacted through Avahan outreach services at least once and 45,000 (129%) had visited Avahan STI clinics at least once by 2009 (Figure 2a).

Monthly FSW contacts by Avahan peer educators and monthly FSW visits to Avahan clinics (Figure 2b) more than doubled between 2005 and 2009. By March 2009, the proportion of estimated FSWs contacted monthly reached 79% and FSWs visiting clinics at least monthly reached 20% (Figure 2b).

Analysis of IBBA data showed an increasing trend for reported lifetime and recent Avahan program exposure. FSWs reporting ever contacted by peer educators increased from 57% to 87% (AOR: 4.5; p<0.001) and FSWs reporting having ever visited Avahan clinics increased from 57% to 86% (AOR=3.9; p<0.001) between Rounds 1 and 2. Similarly, the proportion of FSWs who reported having been contacted by Avahan program peer educators in the last month increased significantly from 54% to 86% in Rounds 1 and 2 respectively (AOR=4.7; p<0.001).

#### Intensity of coverage

**Figure 3:**
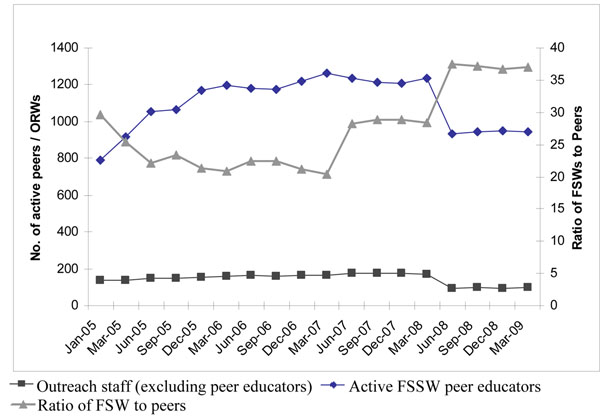
Number of active peers educators / outreach workers and ratio of FSWs to peers educators (Avahan CMIS 2005-2009)

The number of active peer educators and outreach workers was adjusted to maintain optimal levels according to program standards [25]. The average ratio of peer educators to FSWs was about 1:24 until 2007 and increased to 1:35 or higher after March 2008 (Figure 3).

#### Condom distribution, need and availability

Condom distribution by the Avahan program in Tamil Nadu increased sharply from about 2 million in 2005 to about 10 million in 2008. During the same period, the volume of condoms distributed to condom social marketing outlets in Avahan districts increased from over 400,000 in 2004 to about 3 million in 2008. The ratio of condoms distributed monthly by Avahan, per FSW covered by the program increased from 7 (2005) to 25 (2007). The estimated number of monthly commercial sex acts per FSW based on IBBA was 26 in 2006 and 23 in 2009, indicting that sufficient condoms were distributed by the project to cover estimated commercial sex acts by 2008. The source of last condom obtained by FSWs reported in the IBBAs indicated an increasing trend for having received condoms from Avahan; from about 38% to 72% in Rounds 1 and 2 respectively (p<0.001).

**Figure 4:**
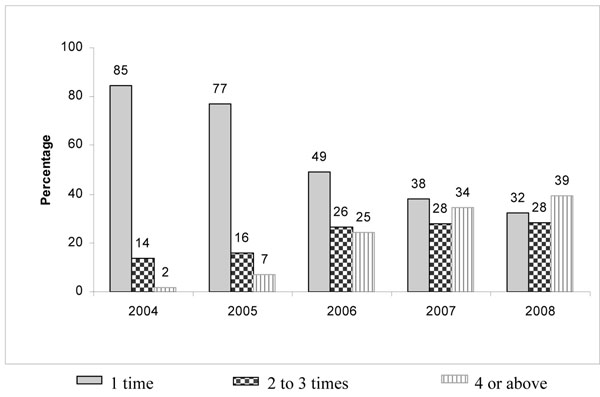
Frequency of visits by FSWs in Avahan program clinics in Tamil Nadu (Individual STI clinic data: 2005-2008)

IBBA data showed that the proportion of frequent FSW monthly contacts (three or more) by peer educators increased between IBBA Rounds 1 and 2 from 33% to 38% (p<0.001) while the proportion of FSWs reporting not being contacted in the last month declined from 7% to zero. MIS clinic data showed that the proportion of FSWs who visited Avahan STI clinics four or more times per year increased five-fold between 2005 and 2008, while the proportion of FSWs who visited only once declined to less than half during the same time period (Figure 4).

#### Quality of coverage

Annual STI clinic quality monitoring assessments covered an average of five established Avahan clinics, which were scaled up from 39 to 51 static and referral clinics between 2005 and 2008. These revealed a consistent increase in quality scores between 2005 and 2008 for clinic performance (1.8 to 3.8; p=0.23), clinic operations (3.6 to 4.9; p=0.12) and clinical management of STIs (3.7 to 4.9; p=0.26). The total aggregate score increased from 3.0 (2005) to 4.5 (2008), (p=0.2) out of maximum score of 5.0. However this increase was not found to be statistically significant.

### Increase in condom use among FSWs

Patterns of reported condom use among FSWs significantly improved between IBBA Rounds 1 and 2. The reported number of no unprotected sex acts increased significantly (AOR=3.8; p<0.001) between two rounds (Table 3). Similarly, consistent condom use with occasional (AOR=5.5; p<0.001) and regular clients (AOR=4.3; p<0.001) increased significantly. Consistent condom use with regular partners continued to be low, and did not change significantly between the rounds (Table 3).

**Table 3:** Changes in intermediate condom use outcomes and HIV and STI prevalence among FSWs in Tamil Nadu

	IBBA R1 N=2032	IBBA R2 N=2006	Crude OR (95% CI)	Adjusted OR (95% CI)	P value (Wald test)
**No reported unprotected sex with commercial clients**	66.7	87.4	3.4 (2.6 – 4.6)	3.8 (2.8 – 5.2)	< 0.001
**Condom use last sex act occasional clients**	92.8	98.1	4.0 (2.3 - 7.0)	5.2 (2.9 - 9.5)	< 0.001
**"Every time" condom use with occasional clients**	72.5	93.1	5.1 (3.6 - 7.3)	5.5 (3.9 - 7.9)	< 0.001
**Condom use last sex act regular client**	90.5	96.1	2.5 (1.7 - 3.9)	3.0 (1.9 - 5.0)	< 0.001
**"Every time" condom use with regular clients**	68.3	89.1	3.8 (2.8 - 5.1)	4.3 (3.1 - 5.9)	< 0.001
**Last time condom use with regular partner**	25.9	21.7	0.8 (0.5 – 1.2)	0.5 (0.4 - 0.8)	0.004
**"Every time" condom use with regular partners**	11.5	16.6	1.5 (0.9 – 2.5)	0.9 (0.6 - 1.5)	0.795
**HIV prevalence**	6.1	6.1	1.0 (0.6 - 1.7)	0.6 (0.4 – 1.0)	0.07
**Syphilis infection**	9.7	2.2	0.2 (0.1 - 0.3)	0.2 (0.1 - 0.3)	< 0.001
**High-titre syphilis**	1.1	0.6	0.6 (0.2 - 1.9)	0.6 (0.2 - 1.9)	0.39
**Chlamydia infection**	2	1.4	0.7 (0.3 - 1.4)	0.7 (0.4 - 1.5)	0.37
**Gonorrhoea infection**	0.5	0.2	0.3 (0.1 - 1.5)	0.5 (0.1 - 1.9)	0.28

Multivariate models were adjusted for the following variables: district, typology, age, literacy, marital status, additional income, sex work for less than 2 years in city and duration in sex work

### Reduction in STIs and new HIV infections

There was a significant decline in the prevalence of lifetime syphilis between IBBA Rounds 1 and 2. This decline in lifetime syphilis was seen in four out of the five IBBA districts (Table 4). The declines in the prevalence of gonorrhoea or chlamydia were not significant (Table 4).

**Table 4:** District-level HIV and syphilis prevalence among FSWs in Tamil Nadu

	IBBA R1 N=2032	IBBA R2 N=2006	Crude OR (95% CI)	Adjusted OR (95% CI)	P value (wald chi-square test)
**HIV prevalence**					

**Chennai**	2.2	2.4	1.1 (.33 - 3.5)	1.9 (.52-7.5)	0.31
**Coimbatore**	6.3	6.3	1.0 (.43 - 2.3)	1.2 (.54-2.8)	0.62
**Dharmapuri-Krishnagiri**	12.4	8.8	0.68 (0.33 –1.4)	0.42 (0.18 -0.95)	<0.05
**Madurai**	4.3	8.3	2.0 (0.75 - 5.5)	1.8 (0.68-4.8)	0.23
**Salem**	12.5	6.7	0.50 (0.25 - 1.0)	0.78 (0.38-1.6)	0.52

**Syphilis prevalence**					

**Chennai**	11.3	0.82	0.06 (.02 - 0.19)	0.05 (.01-.19)	<0.001
**Coimbatore**	11.9	0.37	0.03 (.01 -. 09)	0.03 (.01-.13)	<0.001
**Dharmapuri-Krishnagiri**	10.7	4.2	0.37 (0.17 -0.81)	0.31 (0.11 –0.85)	<0.05
**Madurai**	11.1	2.4	0.19 (0.06 - 0.66)	0.11 (0.02-0.47)	<0.01
**Salem**	7.5	3.1	0.39 (0.15 - 1.0)	0.38 (0.11-1.2)	0.12

Multivariate models were adjusted for the following variables: district, typology, age, literacy, marital status, additional income, sex work for less than 2 years in city and duration in sex work

HIV prevalence among FSWs in the five districts combined, remained unchanged between IBBA Rounds 1 (6%) and 2 (6.1%) (Table 3). In Dharmapuri-Krishnagiri district there was a significant decline in HIV prevalence (12.4 to 8.8; p<0.05) between rounds (Table 4). HIV prevalence declined among young FSWs, aged 18 to 20 years, (5.9% to 0.0; p=0.231) and among those who were new to sex work, <1 year duration, (5.4 to 3.4; p=0.355) declined, but was not significant.

### Association of Avahan exposure with change in condom use and STI prevalence

Consistent condom use and presence of any STIs were examined according to exposure to Avahan program services reported in the IBBA. Logistic regression using pooled data from Rounds 1 and 2 revealed that exposure to Avahan services was associated with consistent condom use with each type of partner (Table 5). However, there was no significant difference in the prevalence of any STIs among FSWs who reported exposure to Avahan and those who did not (Table 5).

**Table 5:** Association of Avahan program exposure with condom use outcomes and having any STI among FSWs in Tamil Nadu

Condom use	Received any one service	Crude OR	Adjusted OR	P value
	
		(95% CI)	(95% CI)	
		
				(Wald test Adjusted OR)
**Consistent condom use with occasional clients**	**78.30%**	2.085**	**1.822**	**p<0.001**
		(1.601-2.715)	(1.350-2.625)	

**Consistent condom use with regular clients**	**80.30%**	2.258**	**2.282**	**p<0.001**
		(1.770-2.880)	(1.781-2.923)	

**Consistent condom use with regular partner**	**13.10%**	3.144**	**2.005**	**0.002**
		(1.913-5.165)	(1.313-3.217)	

**Zero-unprotected sex**	**81.40%**	1.929**	**1.997**	**p<0.001**
			
		(1.472-2.529)	1.503-2.652	

**STI prevalence**				

**Any STI (NG, CT or High-titre syphilis)**	**2.20%**	**0.548**	**0.644**	**0.165**
			
		(0.312-0.965)	(0.345-1.199)	

**<0.01 *<0.05. Multivariate models were adjusted for the following variables: age, district, literacy, marital status, additional sources of income, duration in sex work, typology, sex work less than 2 years in city, round.

## Discussion

This paper presents an assessment of the Avahan program for FSWs in Tamil Nadu using both survey and program data to systematically assess achievements along the project logic model. The analysis provides evidences for increased coverage of the Avahan program, increased condom use with commercial partners of FSWs, decline in STIs and positive association of Avahan exposure to the increased condom use.

Examination of routine program monitoring data revealed that Avahan program in Tamil Nadu was scaled up between 2005 and early 2009, and monthly coverage of 79% (target of 80%) was achieved. The indicators ‘ever contacted’ and ‘ever visited’ program clinics counted FSWs who were contacted or visited clinics at least once. Since these indicators increased well over 100%, it suggests that there is considerable mobility and turnover of FSWs in the coverage districts. IBBA data showed that the coverage of FSWs contacted in the month preceding the survey increased significantly between Rounds 1 and 2, consistent with the CMIS data. This gives confidence that data from the CMIS likely reflects actual coverage, even in Avahan districts where IBBAs were not conducted.

The desired targets for personnel and infrastructure indicators such as ratio of FSWs to peer educators were achieved indicating that the program was scaled up with sufficient intensity by July 2008. The decline in the number of active peer educators after April 2008 was due to a revised strategy by the program to increase the ratio of peer educators to FSWs from 1:30 to 1:35. While some districts were transferred to NACO (Chennai) and some to Avahan (Thiruvallur) in April 2008 as part of the state implementation of phase III of the national program, the Avahan transition of districts to NACO for direct implementation of the established program began after March 2009.

Assessment of condom availability indicated increase in project-supported condom provision and, adequate condoms were available to cover all commercial sex acts of FSWs in Avahan areas by 2008 [19], and this was validated through IBBA data. These data indicate the minimum availability of condoms. Other evidence supports that sufficient condoms were also available through social marketing efforts [26,28] and advocacy [29]. Local BSS data show that over 90% of FSWs reported no difficulty in obtaining condoms [29].

Improved uptake of services was indicated by increasing frequency of contacts and clinic visits. The analysis also revealed that the quality of STI services improved consistently during program implementation. The quality of Avahan coverage was also shown through increase in condom availability through social marketing efforts [26].

IBBA data provided evidence for increased consistent condom use with both occasional and regular clients and a decline in risky sex acts (without condoms) with commercial partners during program implementation. Data on condom use with commercial partners (clients) from other BSS studies in Tamil Nadu also indicate similar trends [29]. The present analysis however found that condom use with regular partners did not increase over time, suggesting the need for more focused efforts in this area. Studies in India and elsewhere have reported low levels of condom use with regular partners, due to issues of trust and intimacy, power relationships, self-efficacy, and fears of partner refusal and violence [30-34].

A significant reduction in some STIs and non-significant declines in other STIs after controlling for confounding factors indicate Avahan’s potential impact. Studies among FSWs in Tamil Nadu in 2004, (prior to Avahan) reported higher prevalence of syphilis (15.7%), gonorrhoea (1.4%) and chlamydia (18.6%) than found in the IBBAs [35]. While these pre-Avahan studies were not conducted in the IBBA districts, Avahan’s STI service package (which includes treatment of symptomatic and asymptomatic FSWs as well as monthly screening) [23] and the delay in the first round of the IBBA (14 months after the start of the intervention), suggests that there may have been an Avahan-related effect. STI services have been shown to be effective in controlling STIs among FSWs elsewhere [11]. Reza-Paul et al (2008) showed a similar reduction in the prevalence of syphilis, chlamydia and gonorrhoea after a shorter duration of intervention (e.g., the first round of the IBBA was done only after six months of intervention) in a Karnataka district which is also supported by Avahan [36,37].

Data are inconclusive about the reduction in new HIV infections among FSWs. Due to the lack of incidence data, HIV prevalence between the two IBBA rounds was examined. No overall change in prevalence in five districts was detected between IBBA rounds. Similarly, no significant change in HIV prevalence was seen in new or young sex workers. Yet, after controlling for confounding, significant declines or stabilization were seen in IBBA districts such as Dharmapuri and Salem (which had no interventions prior to Avahan). The increased HIV prevalence in Madurai district between the two rounds however was not found to be significant. As it is seen here, such increases in HIV have been seen in other Avahan states, though the prevalence of STIs such as syphilis has declined significantly [18]. An increasing trend in HIV among FSWs in Madurai has also been observed from Sentinnel Surveillance in Madurai [38,39] and personal communication from A. Elangovan, NIE]. Additional studies and investigations are required at the district and state level to better understand the factors influencing these trends. Consistent with these trends, results from sentinel surveillance surveys between 2005 and 2008 among FSWs in Tamil Nadu show HIV prevalence to be between 5.5% and 4.5% and stable in most of the 11 districts where data are available [38-40] and personal communication from A. Elangovan, NIE]. Potential reasons for such findings are likely due to high level of condom use and fewer new infections among FSWs. Further, increasing numbers of people enrolling in anti-retroviral treatment (ART) centres in Tamil Nadu and those commencing ART has also increased steadily since 2004 [39,40] may have contributed to this finding.

With respect to association of Avahan exposure with changes in outcomes in Tamil Nadu, there is evidence for increasing consistent condom use with all types of partners among FSWs exposed to Avahan. Yet a definitive causality cannot be established due to limitations of cross-sectional data. Evaluation studies of other peer-mediated interventions outside India as well as those implemented by Avahan in Karnataka have shown similar results [41,42]. However, we found no significant association between Avahan exposure and STI prevalence although evidence of association has been shown by other states implementing Avahan [42,43].

Tamil Nadu has experienced declining HIV prevalence among the general population as indicated by sentinel surveillance data among women visiting antenatal clinics (ANCs), from 1.13% in 2001 to 0.25% in 2006 [38-40] and further to 0.25% (0-3.25%) in 2008 [based on sentinel surveillance data; personal communication from A. Elangovan, NIE]. This has been confirmed by the National Family Health Survey 3 which estimated HIV prevalence of 0.39% among women and 0.27% among men in 2005-2006 [44]. Furthermore, HIV prevalence among pregnant women aged between 15-24 years, a proxy for new infections, estimated from sentinel surveillance dropped from 0.5% in 2005 to 0.32% in 2008. Data on over 900,000 pregnant women attending prevention of parent-to-child transmission (PPTCT) clinics in 2008-2009 showed low HIV prevalence (0.20%) in 2008 [40,45]. Other than the national mid-term review by NACO [46] there has been no systematic evaluation to assess the impact and effects of the HIV prevention in Tamil Nadu.

While the scope of this assessment was not to assess impact of Avahan at the general population level, initial examinations suggest potential contributions. Compared to other districts, Avahan districts had greater risk factors for HIV [47] and, at the start of the project in 2003, had higher HIV prevalence (1.01%) among ANC attendees compared to all districts in Tamil Nadu (0.75%). In 2008, ANC HIV prevalence in Avahan districts was 0.39%, similar to all state districts combined (0.37%). This declining trend was confirmed when examining PPTCT data [39,44] which is generally accepted in India as a proxy for ANC attendees [48]. Analysis of ANC data in Karnataka by Moses et al (2008) indicates a significant decline in HIV prevalence among ANC attendees in Avahan districts compared with non-intervention districts [43]. Tamil Nadu was the first state to detect HIV cases; to initiate a rapid prevention response; and has long-standing history of targeted interventions with multiple players since the early 1990s [38] and these are likely factors contributing to declining HIV prevalence.

While social desirability bias is a common concern in behavioural surveys, we found internal consistency in the self-reported responses for different condom use questions in the IBBAs. Additionally, trends in condom use with different partners between Rounds 1 and 2 were consistent with each other and with studies among clients of FSWs in Tamil Nadu which reported increase in consistent condom use from 40% (2006) to 79% (2008) [28] and provided confidence about the validity of our findings.

A limitation of the assessment is the lack of good baseline data, since IBBA Round 1 was conducted in 2006, nearly 14 months after Avahan implementation. Since the Avahan evaluation did not include control groups, it is not possible to attribute outcomes directly to Avahan interventions. Further, limited data from other non-Avahan districts on outcomes as well as intervention exposures among the FSWs makes it challenging to make other analyses comparing Avahan and non-Avahan areas. While attempts were made to look at unexposed FSWs in Avahan districts, the proportion of these was rather low in Round 2 limiting the power of the analysis.

This assessment was based on the practical considerations and complex conditions account for the highly mobile nature of FSWs with consequent diffusion, explicit intent to scale-up rapidly, and intent to transition programs to government; making it difficult to use government programs as controls [16]. Accordingly, an approach suitable for assessment of large-scale interventions was found appropriate and used here [49-52]. Given these considerations, the strength of the current analysis is that it provides early evidence for effectiveness according to the program logical framework and has showed ‘congruency of expected trends’ [17].

## Conclusions

The findings from the analysis provide strong evidence of successful scale-up and high coverage of the Avahan intervention among FSWs across the implementation districts in Tamil Nadu according to the program logic model within four years of program implementation. High levels of service uptake by FSWs were shown in monitoring data and validated using independent survey data. The intermediate outcome of condom use with clients increased and was found to be significantly associated with Avahan program exposure, with declining trends of syphilis and stabilized HIV prevalence among FSWs. The declining HIV prevalence among the general population groups suggests an effect of the all the programmatic efforts and may be confirmed through future modelling efforts, integrating ANC data and local coverage data from Government programs.

## Competing interests

The authors declare that they have no competing interests.

## Authors’s contributions

TS contributed to conception, design, writing and finalization of the manuscript. BK and VI were involved in design, statistical analysis and interpretation of data. GK was involved in the management of biological component and review of manuscript. AS, RS and CE were involved in implementation and monitoring for data acquisition as well as compiling the manuscript. LR contributed to conception, design, analysis, writing and finalization of manuscript. GT was involved in compiling and review of manuscript. RA was involved in conceptualization, design, review of analysis and final manuscript. RP was the principal investigator for the studies and involved in finalization of manuscript. All authors read and approved the final manuscript.
